# Evaluation of the UK Public Health Skills and Knowledge Framework (PHSKF): implications for international competency frameworks

**DOI:** 10.1186/s12889-020-09024-6

**Published:** 2020-06-18

**Authors:** Anna Bornioli, David Evans, Claire Cotter

**Affiliations:** 1 Centre for Public Health and Wellbeing, Department of Health and Social Sciences, Faculty of Health and Applied Sciences, University of the West of England, Bristol, England; 2 Workforce Development, Public Health, Bristol, England

**Keywords:** PHSKF, Public health, Competency framework

## Abstract

**Background:**

The value of competency frameworks for developing the public health workforce is widely acknowledged internationally. However, there is a lack of formal evaluations of such frameworks. In the UK, the Public Health Skills and Knowledge Framework (PHSKF) is a key tool for the public health workforce across the UK, and this study presents the evaluation of the PHSKF 2016 version, with the aim of reflecting on implications for international public health competency frameworks.

**Methods:**

A sequential explanatory design was employed. An online survey (*n* = 298) was completed with stakeholders across the four UK nations and different sectors. This was followed by 18 telephone interviews with stakeholders and survey completers. Quantitative results were analysed descriptively; qualitative transcripts were analysed with thematic analysis.

**Results:**

Most respondents had used the PHSKF occasionally or rarely, and most users found it useful (87%) and easy to use (82%). Main purposes of use included team/workforce development (e.g. setting of standards) and professional development (e.g. identify professional development opportunities). Some positive experiences emerged of uses of the PHSKF to support organisational redevelopments. However, 23% of respondents had never used the framework. Areas for improvement included greater clarity on purpose and audience, the need for more support from employers and for clear career progression opportunities, and stronger links with other competency frameworks.

**Conclusions:**

The development of a digital version of the PHSKF, together with improving buy-in from the workforce and employers could make an important contribution towards UK public health workforce development. Further evaluation and shared learning internationally of the implementation of public health competency frameworks would support global public health workforce development.

## Background

The scale of global public health challenges requires a knowledgeable and skilled public health workforce [1]. One widely valued approach to support the development of the public health workforce is the construction and dissemination of national or regional (e.g. European) public health competency frameworks to inform public health academic programmes and continuing professional development (CPD). A range of competency frameworks have been developed, in particular in Europe, North America and Australasia [2–9]. Although expressed in different terms, these frameworks are based on a commonly identified need “for a simple tool that facilitates the development of excellence, collaboration and consistency, taking into account the vast diversity of the workforce and varied public health infrastructures and systems that exist.” [9] To date, however, few of these frameworks have been formally evaluated. A search on Medline, Embase, Ovid Emcare, Health Management Information Consortium, PsycInfo and Social Policy and Practice databases identified only three published peer-reviewed evaluations of public health competency frameworks, [10–12] two of which related to the 2016 UK Public Health Skills and Knowledge Framework (PHSKF) [10, 11]. Shickle and colleagues conducted 15 qualitative group interviews with 51 public health practitioners in eight local authorities and found the PHSKF was viewed positively but no participants had previously read or utilised it [10]. Viens and Vass asked two questions about the PHSKF in a wider survey on public health ethics and found that only 38.4% of respondents reported accessing the PHSKF and only 13.7% accessed the accompanying background paper on ethical public health practice [11]. Further searching identified descriptive papers on the development of competency frameworks, but evaluations remain scare.

The lack of evaluations of these competency frameworks is unsurprising as many of them are recent, funding for such evaluation may not be prioritised, and identifying and applying appropriate outcome measures may be methodologically challenging. In particular, as we ourselves found (see Limitations below), the diverse and ill-defined nature of the public health workforce means that the sampling frame for quantitative studies is difficult to identify. Nevertheless, substantial effort and resources go into developing competency frameworks, and public health education and CPD are crucial for an effective workforce, so there is a strong imperative to evaluate their impact, and for learning to be shared internationally. It is notable that to date there have been few published international comparisons of the content or application of the different frameworks. Exceptions are one study validating public health competencies across a range of mainly low and middle-income countries [13], and a comparison of European public health workforce competencies frameworks that is currently in press [5].

In the UK, the Public Health Skills and Career Framework (PHSCF) [14] was first introduced in 2008 with the support of the public health agencies of the four UK nations. This original framework was structured around nine levels of competence and skills, derived from the Key Elements of the Skills for Health Career Framework [15], and nine areas of the UK Faculty of Public Health (FPH) curriculum [16]. In 2015, Public Health England (PHE) led an extensive consultation on this original framework which resulted in clear expressions of the need to simplify and condense the presentation and content [17]. The result was a significantly simplified framework (the PHSKF) that shifted the focus to function and capability descriptors, rather than competencies, that was published in 2016 by PHE on behalf of the public health agencies of all four UK nations [2]. The current study reports results of the evaluation of the 2016 version of the PHSKF. The aim of this evaluation, commissioned by PHE, was to determine the impact of the re-designed framework on the workforce and their employers and its utility; key research questions asked about its use, usefulness, ease of use and extent of use by individuals, teams and organisations. While the evaluation focused on the UK context, several implications emerged that are relevant for the international context and the field of public health competency frameworks. The current paper critically discusses such reflections.

## Methods

The methodology entailed a sequential explanatory mixed-methods design, including an online survey and telephone interviews. Data collection ran from February to March 2019. Ethical approval was given by the University Research Ethics Committee.

An online survey, developed for this study, was built on the Qualtrics platform (available as Supplementary Material). The approach followed a purposeful sampling strategy, aimed at identifying and selecting information-rich cases for the most effective use of limited resources. As the UK public health workforce is diverse and employed in numerous different sectors and organisations, with no central register or listing [18], it was not possible to construct a sampling frame. For this reason, the link to the online survey was circulated by email to a wide range of UK stakeholders from the research team’s network. These included managers and organisations known to PHE to have made use of the PHSKF and a number of additional public health bodies. The request was to widely disseminate it to all those who might be using the PHSKF, including individual public health managers (those responsible for leading teams), specialists (those working at a strategic level or high level of technical expertise), practitioners (those working at an operational or delivery level), those responsible for workforce development, and academics using it for curriculum development. A reminder email was sent one or 2 weeks after first contact. The survey was piloted by several members of the PHSKF Steering Group and university staff.

The survey was followed by in-depth semi-structured telephone interviews with two groups: (a) key stakeholders and (b) a selection of survey respondents who volunteered to give further information. Key stakeholder contacts were provided by PHE and included individuals who had been previously involved in the development of the PHSKF. Survey respondents were purposively selected according to geographical criteria – e.g. to include four respondents from each UK nation – and public health role – e.g. to include individuals working in local authorities, the National Health Service, universities, third sector and the private sector. Although stakeholder views may have been known within PHE, the researchers were blind to both stakeholder and survey participant views when selecting them to the above criteria. Interviews explored in more depth the experiences of respondents in using the PHSKF, the potential impacts that such use had for their work and their organisation, and their views on further developments of the PHSKF, including the potential to develop a digital version. Interviews were conducted between February and March 2019 by the three members of the research team, and ranged between 30 min and 1 h. These were audio-recorded and transcribed with the support of a transcription software.

Survey data from closed questions were analysed with descriptive statistics. Qualitative data from the survey open-ended questions and from the interviews were analysed with thematic analysis [19]. This involved familiarisation with the transcript, development of codes, and development of final themes. Each researcher elaborated notes from each interview, and initial themes were then discussed among the research team. Final themes were agreed among researchers.

## Results

Two hundred and ninety-eight completed surveys were returned and eighteen interviews completed. Table 1 summarises participants’ characteristics.

**Table 1:** Participants’ characteristics

	Survey (%)	Interviews (n)
**Geographical location**		
England	64	8
Northern Ireland	2	2
Scotland	26	4
Wales	6	3
Working Across more than one nation	2	1
**Workplace**		
Local authorities	41	5
NHS trust or health boards	22	3
National public health agencies	19	6
Universities	10	2
Other		2
**Level of public health**^**a**^		
Practitioners^b^	38	5
Specialists	17	3
Managers	18	13
Other	27	1

Note: ^a^Participants had the option to select multiple levels of public health

^b^Including non-registered specialists, advanced practitioners, and practitioners

### Use of the PHSKF

Among individuals who have used the PHSKF, most respondents reported an occasional or rare use, with slight differences among sectors (Table 2).

**Table 2:** Frequency of use by sector

Frequency of use	Total sample	Local authorities	Public health agency	National Health Service	University	Other sector
Regularly (%)	13	11.6	9.4	13.5	20.0	12.5
Occasionally (%)	32	39.1	25.0	40.5	20.0	37.5
Rarely (%)	25	18.8	28.1	27.0	35.0	18.8
Once (%)	7	15.9	9.4	0.0	0.0	0.0
Never (%)	23	14.5	28.1	18.9	25.0	31.3
*n*	*254*	*69*	*32*	*37*	*20*	*16*

The most important use was independent (e.g. personal professional development), but specialists tended to use the PHSKF as group leaders (e.g. for workforce development) (Table 3)

**Table 3:** Most important use by level of work

Use of the PHSKF	Answer	By total sample (%) (*n* = 149)	By level (%)			
			Specialist (*n* = 37)	Non-registered specialist or advanced practitioner (*n* = 38)	Practitioner (*n* = 31)	Manager (*n* = 31)
Type of use	*Independent*	45.0	32.4	42.1	67.7	33.3
	*As part of team activity*	19.5	8.1	26.3	12.9	24.4
	*As group leader*	20.8	45.9	18.4	6.5	24.4
Purpose	*Personal professional development*	42.2	27.0	39.5	61.3	30.3
	*Team development*	12.2	13.5	7.9	6.5	21.2
	*Teaching*	10.2	8.1	15.8	3.2	3.0
	*For workforce development*	24.5	37.8	26.3	22.6	30.3
Specific tasks	*To identify CPD opportunities*	16.3	10.8	21.1	13.3	9.1
	*Assessment against criteria*	16.3	2.7	13.2	30.0	3.0
	*To plan/develop curriculum*	9.5	10.8	13.3	0	0
	*To write job descriptions*	9.5	29.7	5.3	3.3	3.0
	*Setting of standards*	8.2	8.1	5.3	6.7	15.2
	*To carry out a team skills audit*	7.5	10.8	7.9	3.3	15.2
	*In appraisal review*	7.5	5.4	5.3	16.7	3.0
	*Other*	25.2	21.7	28.6	26.7	51.5
n		149	37	38	31	31

### Strengths

Most respondents found the PHSKF useful, easy to use, and reported a positive impact (Table 4). However, from interview discussion it emerged that it is still early to assess the impact of the use of the framework.

**Table 4:** Usefulness, ease of use, and impact of the PHSKF

	*Extent to which PHSKF is:*			Impact generated by PHSKF (%) ***n*** = 149
	Useful (%) ***n*** = 147	Easy to use (%) ***n*** = 148		
*Strongly agree*	18	12	*Extremely positive*	12
*Agree*	43	44	*Moderately positive*	47
*Somewhat agree*	27	27	*Slightly positive*	23
*Neither agree nor disagree*	4	5	*No noticeable impact*	17
*Somewhat disagree*	6	9	*Negative impact*	1
*Strongly disagree*	2	3		

The two most important uses of the PHSKF are around personal professional development and workforce development. Regarding the former, this includes, for example, assessment against the criteria and identification of CPD opportunities:

*I used the PHSKF to identify specific learning needs. After doing this I was able to apply for a more senior position in the department (Survey respondent 214).*

Turning to workforce development, the PHSKF has assisted some organisations in their redevelopment. An examplar case is the staff development program implemented by Lincolnshire County Council in 2016. This assessed individual knowledge, skills strengths, gaps and development needs based on the PHSKF, which then formed the basis for staff training programmes:

*We identified training needs, and we used that to plan our CPD and training programmes and map it to the framework. When people go to a CPD session, the agenda will say ‘mapped to areas in the framework’. That’s very helpful, because this training will start to bridge [identified gaps]. (Interviewee 7).*

### Limitations and barriers to use

Twenty-three percent of respondents had never used the framework, due to not having knowledge around it (47%) or no need to use it (31%). The qualitative analysis identified barriers to use and limitations of the PHSKF. First, it emerged that there is some uncertainty around the aims, focus, and audience of the PHSKF:

*I don’t think that that’s really out there. […] I don’t think it’s well known what the benefits of that could be (Interviewee 10).*

Target audience is one specific area of uncertainty, with some respondents unsure whether it applies to all public health professionals and levels:

*Is this supposed to cover up to specialist level? That is a massive breadth of practice. That’s a bit like in education trying to have a curriculum that covers everything from GCSE up to doctorate. […] (Interviewee 15).*

Second, respondents noted a perceived lack of formal recognition in formal processes at both organisational and national level, and lack of ‘buy-in’ from managers:

*At present PHSKF very much feels like something one ‘could’, if motivated, use […]. It would be useful to make this an explicit part of yearly appraisals/PDP/objectives [and] of recruitment (Survey respondent 96).*

*There needs to be buy-in from senior managers, [otherwise] it’s quite difficult. And what’s the buy-in from PHE? […] Is there an expectation nationally? (Interviewee 7).*

Third, some respondents do not feel motivated to use the PHSKF because it does not lead to clear career progression opportunities:

*It provides clarity at each ‘step of the ladder’ but it does not really help individuals to climb the ladder. So it is a systems (conceptual) tool but doesn’t directly support individual career progression (Survey respondent 200).*

This is also related to the absence of levels in the new version of the framework, compounded by a lack of perceivable uniformity in workforce levels across a range of employers, which might compromise its utility and turn it into a tick-box exercise:

*I think [the loss of levels] is not so helpful. I can understand it’s a focus on competency, not progression, but the advantage of the other [version] was that you could see how those competencies played out at a certain level of practice, and what you needed to do (Interviewee 15).*

*One could blast through the competencies as a tick-box exercise and say that they have them all (convince themselves even), but fall down when it comes down to actually performing (Survey respondent 96).*

In relation to career mobility, some respondents see the PHSKF and the UK Public Health Registrar (UKPHR) standards (which must be met for practitioners to be included on the UKPHR’s voluntary register of public health practitioners) [20] as alternatives, and will opt for the UKPHR route which is perceived as having a clearer career progression outcome:

*I’m probably going to go down the [UKPHR] scenario as I’m going to get something out of it. And I don’t get anything out of the framework. Is it worth it? Will it be acknowledged by people who are going to potentially employ you or by your employers? (Interviewee 6).*

## Discussion

Whilst there exist numerous public health competency frameworks in the international context, to date there is a lack of evaluations of such frameworks. The current study has identified strengths and limitations of the UK PHSKF; based on the findings, several lessons can be drawn for other international public health competency frameworks.

It emerged that the PHSKF is generally valued by the workforce and perceived as useful. It is used for personal professional development purposes (mainly by practitioners) and for workforce development (by managers and specialists). When employed at organisational level, it has assisted some organisations in their workforce redevelopment, providing excellent guidance to identify skill gaps and opportunities. However, some challenges in practical embedment have emerged. These are mostly related to a lack of clarity on the purpose of the framework and how it relates to career mobility and/or progression opportunities, lack of encouragement for use at local organisational level, and uncertainty on how it relates to other national public health frameworks.

There are several implications for the effectiveness of public health competency frameworks that follow and are relevant beyond the UK context. First, it is key to have a combination of top-down action from national leaders and local managers and bottom-up involvement from the workforce. On one hand it is crucial for managers to encourage the workforce to employ such competency frameworks. This can be done by designing framework-based training programmes – such as in the case of Lincolnshire – or by embedding such frameworks in formal processes, including recruitment and appraisals. At the moment, the use of the PHSKF in the UK is inconsistent and relies on the individual initiative of local managers to implement it within their teams. In order to achieve wider use, a more systematic culture change at national level is needed. On the other hand, there needs to be a strong buy-in from the workforce, and individuals need to see the practical value of using such frameworks for career mobility as well as progression. A potential way of incentivising users is to offer more user-friendly versions of the frameworks, as is planned in the UK with the development of a digital version of the PHSKF.

Second, clarity on purposes and audience is key. In the current study, some individuals were not aware of the existence of the framework, and others were unsure about its purpose, target audience, and links to other national public health frameworks. There appeared to be several possible misconceptions about the intended use of the framework; for example, seeing the PHSKF as an ‘either/or’ choice with registration, or seeing registration as an end in itself in terms of career progression rather than part of continuing professional development which contributes to sustained competence and career management. Our findings echo the two other UK studies which asked about knowledge and use of the PHSKF [10, 11]. We do not know how effectively other competency frameworks have communicated their purposes to intended audiences due to the lack of published evaluations, but the UK experience suggests this may be a key issue the architects of other frameworks should consider.

Third, a concern expressed by informants was the challenge for the PHSKF to cover everyone in the public health workforce from entry level up to senior professionals. This expectation for ‘levels’ to reflect either aptitude or expertise in any given competence, or hierarchical positioning with an organisation or system can create problems for competency frameworks particularly as public health systems are at such different stages of development. The PHSKF redesign also had to support the finding that, in practice, public health workers may operate at varying levels depending on the function, within the same role. Hence the redesigned framework presents a menu of functions that include strategic and operational level descriptors. Based on the workforce profile of survey respondents in the review of the 2008 PHSCF, it was proposed that three workforce levels should be illustrated in the PHSKF, reflecting other competency frameworks [3, 5], and this work is currently being progressed to further support wider adoption of the PHSKF.

Finally, the UK experience suggests the crucial importance of evaluating the use of public health competency frameworks in real world practice. The evaluation of the original 2008 PHSCF led to significant improvements in the 2016 PHSKF [16, 21]. Our evaluation has generated further valuable insight into the strengths and limitations of the PHSKF and highlighted areas for development with the planned future digital version. In our literature search we found published studies on the development and validation of other frameworks [8, 13], but none evaluating the use of other competency frameworks in practice other than one local US study of a competency-based training programme [12]. None of the non-UK national or regional competency frameworks [2–9] we have identified have published evaluations in use. The development of public health competency frameworks internationally would benefit from both further evaluative studies of individual national and regional frameworks and of comparative studies of different frameworks. Moreover, given the scarcity of evaluations of public health competency frameworks, there is a wider literature on the theoretical background to, and the evaluation of, competency frameworks in other professional settings and contexts, which evaluators of public health competency frameworks might usefully learn from [10, 22, 23].

Limitations of the current evaluation include the non-probability sampling frame of the survey and the relatively low response rate, considering that the core public health workforce is estimated to include between 36,000 and 41,000 in England alone [17]. The recruitment strategy was purposeful, given the short time-scale of the study and limited resources, and mainly targeted organisations that were known to PHE to have made some use of the PHSKF. Hence, it is possible that results are not representative of the entire public health workforce but reflect the views of those groups which were more familiar with the framework. Another limitation is that socio-demographic charactersitics of participants were not collected, and these could have added interesting insights to the study. Finally, the geographical sampling criterion was not met in Wales and Northern Ireland due to lower participation rates.

## Conclusions

The current study has evaluated strengths and weaknesses of the UK PHSKF, and represents one of the few evaluations of public health competency frameworks in international contexts. It has highlighted that the PHSKF has potential to generate a positive impact among the UK public health workforce. Several implications for the UK and international contexts emerged. First, it is important to enhance buy-in from managers and workforce; second, clarifications of purpose and target audience of the framework are crucial; and third, development of a digital version and related digital tools could improve the success of public health competency frameworks. Further evaluation of public health competency frameworks are needed to support global public health workforce development.

## Data Availability

The datasets used and/or analysed during the current study are available from the corresponding author on reasonable request.

## Abbreviations

CPD: Continuing Professional Development
FPH: Faculty of Public Health
PHE: Public Health England
PHSCF: Public Health Skills and Career Framework
PHSKF: Public Health Skills and Knowledge Framework
UKPHR: UK Public Health Registrar
